# Diagnostic approach to swinepox virus infection in a German 2-site swine production unit

**DOI:** 10.1177/10406387251366960

**Published:** 2025-08-30

**Authors:** Susanne Richter, Friedrich Schmoll, Daniel Polzer, Christoph Leth, Sandra Revilla-Fernández, Lukas Schwarz, Angelika Auer, Tatjana Sattler

**Affiliations:** Institute for Veterinary Disease Control, AGES, Mödling, Austria; Institute for Veterinary Disease Control, AGES, Mödling, Austria; Institute for Veterinary Disease Control, AGES, Mödling, Austria; Institute for Veterinary Disease Control, AGES, Mödling, Austria; Institute for Veterinary Disease Control, AGES, Mödling, Austria; Clinical Department for Farm Animals and Food System Science, University of Veterinary Medicine Vienna, Vienna, Austria; Department of Biological Sciences and Pathobiology, University of Veterinary Medicine Vienna, Vienna, Austria; Clinic for Ruminants and Swine, University Leipzig, Leipzig, Germany

**Keywords:** electron microscopy, PCR, sequencing, therapy, swine, swinepox virus, thymidine kinase

## Abstract

In 2008, nearly 50% of weaned piglets at a German 2-site production unit in Saxony-Anhalt had skin lesions 1–2 wk after relocation into the nursery. First clinical signs were maculae, followed by papules, pustules, and finally crusts, distributed over the dorsal and lateral body flank. Tentative clinical diagnosis was an infection with swinepox virus (SWPV; family *Poxviridae*, taxon species *Suipoxvirus swinepox*). Electron microscopy confirmed within one hour that the causal agent was a brick-shaped poxvirus, and routine PCR validated the poxvirus detection; PCR for *Orthopoxvirus* was negative. Phylogenetic analysis of the thymidine kinase genes from different poxviruses and from our SWPV isolates, 3 isolates from Germany, and 1 isolate from Austria, provided a good picture of evolutionary relationships of poxvirus genera, which was also consistent with phylogenetic analysis of poxviruses based on other genes. The German and Austrian isolates from domestic pigs were 99.8–100% identical to previously isolated German SWPV from wild boar and domestic pigs. All isolates belonged to the North American/European lineage. In a second step, SWPV assembly in naturally infected domestic pigs was analyzed by ultrathin sectioning. The virus assembly resembled that of other poxviruses and completed gaps in the SWPV morphogenesis model described in prior publications. Because there is no specific therapy, recommended interventions were improvements in biosecurity measures, especially hygiene management and disinfection procedures at the farm and within the transporters between the farrowing unit and the nursery. No further infections with SWPV were seen 5–6 wk after commencement of the hygiene interventions.

Poxviral diseases of pigs can be attributed to swinepox virus (SWPV; family *Poxviridae*, taxon species *Suipoxvirus swinepox*) but also to cowpox virus (CPV; *Orthopoxvirus cowpox*) or vaccinia virus (VACV; *Orthopoxvirus vaccinia*). In Europe, CPV infection and VACV infection of pigs were mainly documented in inoculation experiments^49^ and during smallpox vaccination campaigns,^7^ whereas in Brazil and Asia, VACV infections are endemic.^19,32^ Detection of poxvirus DNA using PCR and differentiation with subsequent sequencing allows final diagnosis of infection with SWPV.

SWPV, the sole member of the genus *Suipoxvirus* in the subfamily *Chordopoxvirinae*,^29,30^ is host specific, and only members of the family *Suidae* become infected. The enveloped virions of genus *Suipoxvirus* are generally brick-shaped with a size of ~300 × 250 × 200 nm.^30^ The virions are morphologically similar to the virions of orthopoxviruses, with surface filaments arranged irregularly in tubular units. The genome is a single, linear molecule of covalently closed dsDNA of ~175 kbp with inverted terminal repeats of ~5 kbp. SWPV is of special interest because it is a promising vector for viral vector-based vaccines for the prophylaxis of many porcine diseases.^52^

Swinepox is an acute, contagious, sometimes pyrogenic infectious disease that is accompanied by papular skin lesions. In pigs <3-mo-old, the clinical diagnosis of congenital SWPV infection is usually easy because of generalized skin lesions^5,36,46,51^ and high morbidity.^15,32,37^ Older pigs have less-pronounced skin lesions.^11,24^ Infections in older pigs occur sporadically, mostly in backyard farms with poor hygienic conditions. The disease, which is a non-notifiable disease by the World Organisation for Animal Health, is generally self-limiting with a low mortality rate, but morbidity can reach up to 100% in cases of poor sanitary conditions, improper husbandry practices such as overcrowding, and ectoparasite infestation.^11,20^ The virus is very resistant and can persist in a dry environment for nearly 6 mo.^19^ The reservoir and source of infection is mainly infected pigs. Hence, in most cases, transmission occurs by direct contact with infected pigs; however, transmission can also occur indirectly by insects [e.g., by pig lice (*Haematopinus suis*), houseflies (*Musca domestica*), or possibly other insect vectors^11,34,46^] and by contaminated farm equipment.^11^

Given that SWPV infections are not very common, several differential diagnoses should be considered, such as infections with bacteria (e.g., *Staphylococcus hyicus*, *Streptococcus* spp., *Bacillus* spp.), infections with other viruses [e.g., porcine parvovirus (PPV), porcine circovirus 2 (PCV2), porcine reproductive and respiratory syndrome virus (PRRSV), foot-and-mouth disease virus (FMDV), vesicular stomatitis virus (VSV)], scabies, dermatosis vegetans, selenium toxicity, allergic reactions, and insect bites.^15,26,34,51^

Our study aimed to present a diagnostic procedure for swinepox, describing the clinicopathologic, molecular, and treatment features of SWPV isolates from Saxony-Anhalt, Germany, and Styria, Austria.

## Materials and methods

### Case description

Between November 2008 and March 2009, piglets in a German 2-site production unit in Saxony-Anhalt had red skin lesions. The farm operates 2 locations, a piglet production unit of ~600 sows, which is located 6 km from the piglet-rearing site and its attached fattening unit. Sows were regularly vaccinated against PRRSV, *Erysipelothrix* sp., and PPV; piglets were vaccinated against *Mesomycoplasma* (*Mycoplasma*) *hyopneumoniae* and PCV2. Fifty percent of weaned piglets developed skin lesions 1–2 wk after relocation into the nursery. Skin biopsies of 7 piglets were analyzed for poxvirus. Red skin lesions developed to the macular stage, followed by papular, vesicular, and pustular stages, before scabbing over and desquamation. Signs first seen 1–2 wk after relocation into the nursery appeared in subsequent weaning groups of the same age. Sows and suckling piglets were not affected. Infections were also not evident in the grower–finisher unit.

### Diagnostic approaches

The first tentative clinical diagnosis was an infection with SWPV after exclusion of other possible animal diseases by routine testing. VSV, FMDV, PCV2, and PRRSV were excluded by PCR, and insect bites by light microscopy. Skin tissue scrapings from crusts, as well as epidermal biopsies, were collected for poxvirus detection. Biopsies of the lesions were analyzed by routine PCR^44^ and transmission electron microscopy (TEM) immediately without preservation; parts of the biopsies, frozen at −20°C, were analyzed later. Frozen biopsies (–80°C) of lesions of suipox-positive newborn piglets from a February 2020 outbreak in an Austrian piglet production farm^46^ were also analyzed to confirm the diagnostic approach and for phylogenetic studies on SWPV.

Routinely, PCR^44^ was used for molecular detection. SWPV-specific PCR primers (forward: 5′-CCGAGGAGTAT-3′; reverse: 5′-AGGAATCCGTAGATACAGCCGA-3′) were designed with the PrimerQuest Tool (Integrated DNA Technologies). The primers were based on published DNA sequences of the hypothetical protein gene (*SPV062*, forward primer: position 55,363–55,386) and the conserved thymidine kinase (*TK*) gene (*SPV063*, reverse primer: position 55,814–55,835) of the North American SWPV strain 17077-99 (GenBank AF410153^1^). These primer sequences are equivalent to sequences of the genes *SwF7* (forward primer: position: 38–61) and *SwF8* (reverse primer: position 491–511) of the North American strain M59931.1.^45^ Orthopoxvirus DNA (CPV DNA) was used as a negative control in the PCR to control specificity. PCR products of the expected size of 473 bp were sequenced and aligned to confirm the identity of SWPV DNA (BigDye Terminator v.1.1 cycle sequencing kit; Applied Biosystems). Sequencing reactions were carried out (PRISM 310 genetic analyzer; Applied Biosystems). The molecular test was completed with an orthopoxvirus-specific PCR. The differentiation from the genus *Orthopoxvirus* is extremely important because infections with orthopoxviruses are notifiable in Germany. Molecular detection of *Orthopoxvirus* was performed according to a published method.^43^

To gain more knowledge about the phylogenetic relationship of the *TK* genes, *TK* genes of various poxviruses in the subfamily *Chordopoxvirinae* were analyzed and compared with the *TK* genes of the recent German and Austrian field isolates. We used the *TK* gene of SWPV, the *SwF8* gene (syn. *SPV063* gene), with its neighboring genes *SwF7* and *SwF8a* (forward primer: 5′-ACGAACCATTATCCGAGGAG-3′, position 26–46, reverse primer: 5′-GGTCCAATCAGGCTTTAACAC-3′, position: 1,042–1,063; PCR product size: 1,037 bp) of strain M59931.1.^45^ DNA was extracted (BioExtract Superball extraction kit; BioSellal) combined with the KingFisher purification system (ThermoFisher) for automated nucleic acid extraction. PCR amplification was performed (Multiplex PCR master mix; Qiagen) in a final volume of 25 µL (0.6 µmol of each mentioned primer, 95°C for 15 min followed by 40 cycles of 94°C for 30 s, 50°C for 45 s, 72°C for 70 s, and a final extension at 72°C for 10 min). The amplified gene fragments were visualized on a 1.5% agarose gel, and PCR purification was performed (QIAquick gel extraction kit; Qiagen). The sequencing PCR was performed (BigDye Terminator v.3.1 cycle sequencing kit; ThermoFisher) followed by a final purification (DyeEx kit; Qiagen) to eliminate unincorporated dye terminators. Capillary electrophoresis was performed (SeqStudio 8 Flex genetic analyzer platform kit; ThermoFisher). Representative *TK* gene sequences of various SWPV isolates and other poxviruses were downloaded from the NCBI database and were used for phylogenetic analysis. The evolutionary analysis was conducted using IQ-TREE 2 (https://iqtree.github.io/) with its “model finder plus” option enabled. The resulting phylogenetic tree was later visualized using the iTOL web tool (https://itol.embl.de/).

TEM of epidermal lesions was performed by negative staining and tissue sectioning. Rapid ultrastructural detection of a poxviral infection after clinical suspicion is usually achieved within one hour. For negative staining, each sample was put into an extraction bag (Bioreba) filled with 4°C-cold PBS buffer (pH 7.3). The relationship of sample to buffer was 1:5 w/v. The sample was ground with a homogenizer hand model (400010; Bioreba). Each biopsy suspension was cleared by low-speed centrifugation before being placed onto carbon-coated grids; the virus-loaded suspension was also sedimented directly onto the grids by ultracentrifugation (Airfuge, 110,407 × *g*, 20 psi; Beckman); 0.5% aquatic uranyl acetate solution or 0.5% phosphotungstic acid were used as negative stains. Biopsy samples were immersion-fixed in cold Karnovsky fixative (2% paraformaldehyde, 2.5% glutaraldehyde, 4°C, pH 7.3), then post-fixed in 1% buffered osmium tetroxide and processed for ultrathin sectioning according to conventional methods. Semithin 70-µm Epon sections stained with Richardson solution were analyzed in a light microscope; ultrathin sections, 70-nm thick, stained with 1% methanolic uranyl acetate and Reynolds lead citrate, were finally examined with a transmission electron microscope (model 906; Zeiss) at 80 kV.

## Results

### Course of infection, therapeutic interventions, and outcome

**Figure 1.**
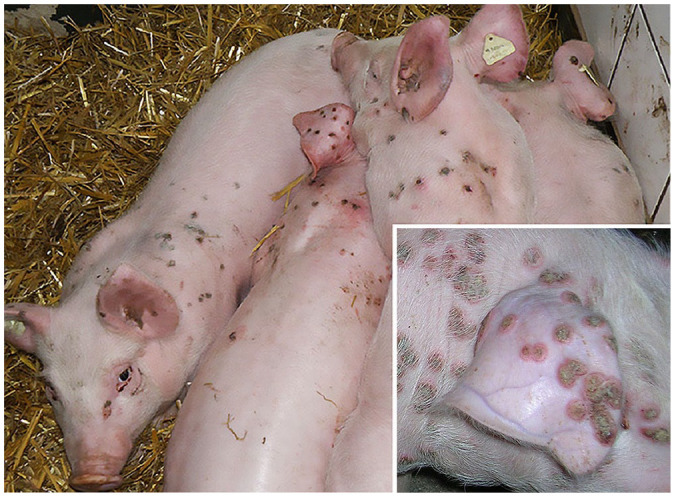
Piglets in close body contact with pox lesions on the head, dorsum, and on the lateral abdomen. Inset: pox lesions on head, ear, and back.

The morbidity rate in weaned piglets was ~50%. The first clinical signs—red 1–2-cm skin lesions in the form of maculae—were observed 1–2 wk after relocation into the nursery. The lesions were mainly located on the lateral flank, back, neck, head, and ears (Fig. 1). Lesions occurred in different forms, as maculae, papules, vesicles, pustules, and crusts. Separate papules often had wall-like borders with depressed centers. In severe cases, papules often merged to form large plaques. Crusts fell from the lesions 4–6 wk after occurrence of the first signs, and recovery proceeded without scar formation.

Because there is no specific therapy against poxvirus, interventions amounted to improvement of hygiene procedures and disinfection methods at both the farm and within transporters from the farrowing unit to the nursery. Pig sheds were cleaned and disinfected after the clinical diagnosis. To prevent secondary skin infections, weaned piglets received antibiotic treatment via drinking water for the first 7 d after arrival at the nursery unit until cessation of clinical signs of infections with SWPV. The treatment prevented secondary bacterial dermatitis and, therefore, a decrease in morbidity in each weaning group. After 5–6 wk, no more clinically apparent infections occurred.

### Molecular approaches

Infection with SWPV was confirmed by routine PCR. All amplified products were 473 bp long and, after sequencing, the gene fragments had 100% identity to published sequences of the European/North American strains (NCBI database: AF410153,^1^ NC_003389,^1^ MZ682626,^13^ MZ773480,^22^ MZ773481,^22^ M64000,^14^ M59931^45^). PCR for orthopoxvirus was negative.

**Figures 2, 3.**
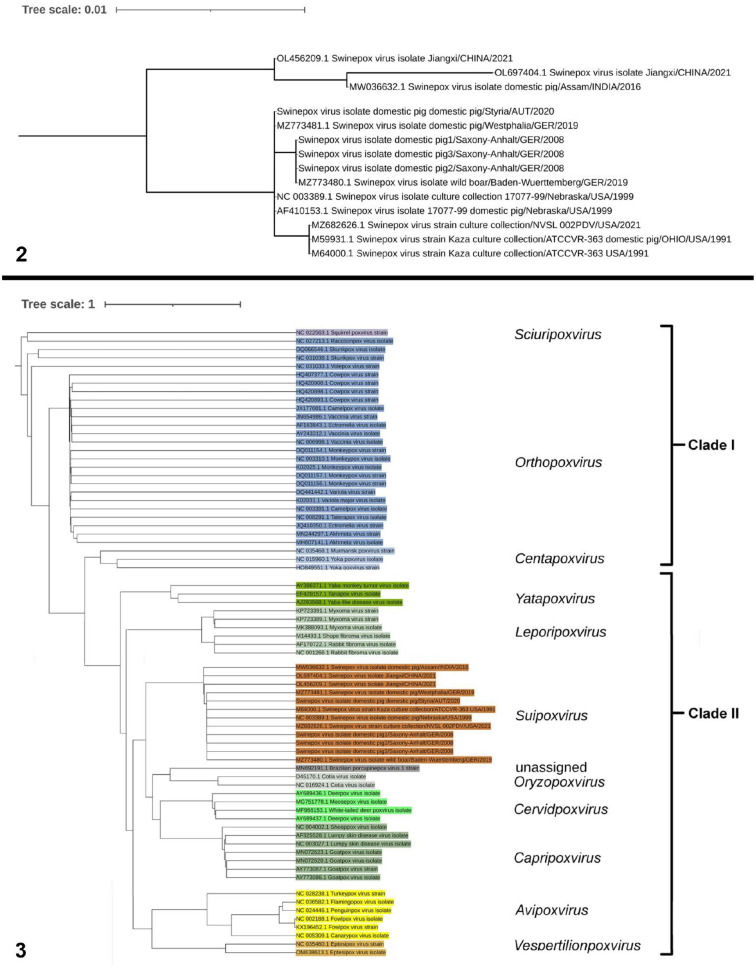
Maximum-likelihood phylogenetic trees. **Figure 2.** Maximum-likelihood phylogenetic tree of the German and Austrian swinepox virus (SWPV) isolates; evolutionary relationship of the thymidine kinase genes of the SWPV isolates of the European/North American lineage and Asian lineage. **Figure 3.** Maximum-likelihood phylogenetic tree based on the thymidine kinase genes of various members of the subfamily *Chordopoxvirinae.* The sequences clustered in 2 clades, clades I and II. Clade II includes the poxvirus group of the CSYLC genera, the *Oryzopox-*, *Avipox*-, and *Vespertilionpoxvirus*. Clade I, which includes the poxviruses of the genera *Orthopox-*, *Centapox*-, and *Sciuripoxvirus*, was more isolated.

Phylogenetic analysis (Fig. 2) of the whole *SwF8*/*TK* gene sequences of different *Suipoxvirus* strains from GenBank revealed the close genetic relationship to the Austrian and other German field strains (German domestic pig strain: MZ773480; German wild boar strain: MZ773481)^22^ and to North American SWPV isolates (domestic pig strains: AF410153 = NC003389.1, MZ682626.1, M64000.1 = M59931),^1,13,14,45^ whereas Asian field strains (Indian field/domestic pig strain: MW036632.1,^3^ unverified Chinese field strains published in GenBank: OL456209.1, OL697404.1) had more sequence differences. Concerning the *TK* gene, the German domestic pig strain from our location in Saxony-Anhalt is more closely related to the German strain isolated from wild boar in Baden-Wuerttemberg (100% homology) than to the Austrian isolate and German isolate of the domestic pig from Westphalia (99.9% homology). Phylogenetic analysis of the *TK* sequence of poxviruses of different genera (Fig. 3) identified 2 clades: clades I and II. Clade I comprised poxviruses in the genera *Sciuripox-*, *Orthopox*-, and *Centapoxvirus*; clade II comprised poxviruses from the genera *Yatapox*-, *Leporipox*-, *Suipox-*, *Oryzopox-*, the unclassified porcupinepox virus, *Cervidpox-*, *Capripox-*, *Avipox*-, and *Vespertilionpoxvirus*.

### Electron microscopy

**Figures 4–6.**
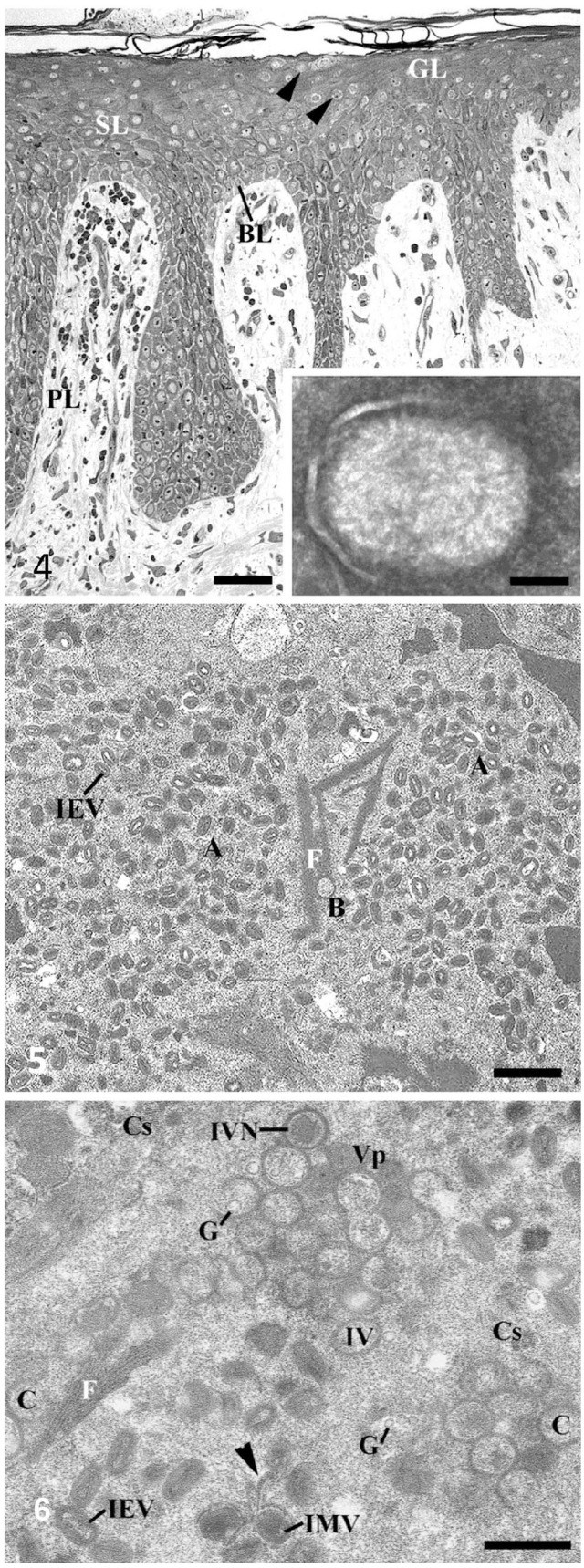
Swinepox virus (SWPV) infection. Transmission electron micrographs. **Figure 4.** Skin lesions from infected pigs. Epidermal hyperplasia with ballooning degeneration (arrowheads) of keratinocytes of the spinous layer (SL) and granular layer (GL), by nuclear vacuolation and by formation of intracytoplasmic inclusion bodies. BL = basal layer; PL = papillary layer of the dermis. Semithin section. Bar = 100 µm. Inset: a brick-shaped extracellular enveloped SWPV, 250 × 350 nm, with a surface membrane of irregularly arranged, 10–30-nm thick tubule elements (= mulberry form of purified virus). Bar = 100 nm. **Figures 5, 6**. SWPV assembly. **Figure 5.** A-type inclusion zones (A) with mature virions and B-type inclusion zones (B) with immature virions and long cross-striated fibrils (F) often form large compact areas that are not clearly delimited from other cytoplasmic regions of keratinocytes. IEV = intracellular enveloped mature virions. Bar = 1 µm. **Figure 6.** Virus factory or B-type inclusion with various stages of virus assembly: the normal sequence of virus assembly can be described as the emergence of membrane crescents (C), the enlargements of these crescents into spherical immature virions (IV) with finely granulated virosome and sometimes with globular elements, the appearance of immature virions with condensed viroplasm (IVN), and the restructuring into mature brick-shaped intracellular virions (IMV). A cisterna of intracellular membranes of unknown origin (arrowhead) is extending around the IMV to form the outer membrane. Finally, the intracellular enveloped virion (IEV) with nucleosome, core membrane, lateral bodies, and outer membrane is formed. Cs = crystalloid deposits in the viral matrix; F = parallel, cross-striated fibrils; G = globular elements; Vp = viroplasm. Bar = 500 nm.

TEM confirmed the clinical diagnosis of poxvirus infection within one hour after sample arrival in the laboratory. In semithin sections, SWPV particle concentration was most significant in the ballooning and reticular degeneration of keratinocytes of the upper zone of the epidermis (Fig. 4). In negative staining, extracellular enveloped SWPV particles (Fig. 4 insert) had an outer virus envelope and a surface membrane with irregularly arranged, 10–30-nm thick tubular elements. The keratinocytes of the spinous and granular layers were often ruptured. As revealed by TEM, reticular degeneration of these keratinocytes was caused by viral proliferation forming large cytoplasmic A-type (A) and B-type (B) viral inclusion bodies full of virions at different developmental stages (Fig. 5). The cytoplasm of infected keratinocytes consisted of a homogeneous matrix rich in ribosomes, vesicles of the endoplasmic reticulum, small Golgi complexes, and mitochondria at various stages of degeneration. A diffuse network of fine fibrils and filaments was dispersed in their cytoplasm. Large central membranous bodies surrounded by mature virus particles, described in other poxviral infections, were not detected in our samples. The A inclusion bodies were filled with intracellular enveloped mature virions, many filaments, and uniformly granular, electron-dense material. Smaller B inclusions, so-called “virus factories,” represented the sites of viral assembly (Fig. 6). The B inclusion bodies contained immature virus stages, from crescent stages to immature virions with condensed viroplasm. The granular matrix of B inclusions included numerous ribosomes, clusters of crystalloid deposits in a lattice-like pattern, and bundles of 0.75–2.6-µm long, parallel, cross-striated fibrils, seen as alternating light and dark bands. The number of cross-striated fibrils was more prominent in B inclusions than in A inclusions. The spacing between the 16-nm thick fibrils was very regular. Immature virions and crescents within the cytoplasm were often in close contact with these straight or gently undulating fibrils. Immature virions developed by accumulation of dense foci of uniform viroplasm surrounded by crescent-shaped membranes later closing to spherical immature virions. Early virus maturation started with viroplasm condensation (“nucleoid pattern”) of immature virions. The contracted viroplasm differentiated to form intracellular mature virus particles, which were enveloped by a cisterna of intracellular membrane of unknown origin to form an intracellular enveloped virion. Intracellular mature virions and intracellular enveloped virions often existed in clusters in A inclusions outside, or on the periphery of, virus factories.

Details of swinepox virus assembly are shown in Suppl. Fig. 1. The earliest evidence of virus assembly was the appearance of rigid crescent-shaped structures (Suppl. Fig. 1A). Crescents, which were single-membrane bilayers with a smooth concave side and an external surface composed of a continuous honeycomb lattice, never had any attachments to membranes of the endomembrane system. They usually ended blindly or in a loop (Suppl. Fig. 1A, asterisk). Occasionally, their free ends were in contact with 15–20-nm globular elements, which were also found in the cytoplasmic granular matrix of B inclusions. Straight or gently undulating fibrils were often in contact to early virus stages (Suppl. Fig. 1B). Early A inclusion formation was often seen as the attachment of intracellular enveloped virions around small vacuoles (Suppl. Fig. 1C) and, in some cases, to areas with crystalloid structures (Suppl. Fig. 1D). Infected cells contained numerous multivesicular bodies (Suppl. Fig. 1E). Compact packages of tonofibrils were often found at the periphery of these epithelial cells close to the plasma membrane. A small number of large lipid globules and numerous bacteria from secondary infections were also detected in the epidermal cells of infected pigs (Suppl. Fig. 1F).

## Discussion

Outbreaks of swinepox are reported sporadically from Europe,^11,22,24,28,46^ Australia,^21^ North and South America,^32,35^ and frequently from Asia.^3,20,31,41^ Special attention has been given to congenital cases, which usually lead to high case-fatality rates.^5,36,46,51^ Transplacental infection of the developing fetus can occur; piglets were born with pox lesions distributed over their entire skin surface, with particularly high-grade ulcerations in the oral cavity. In our case, transplacental transmission was not evident; sows and their suckling piglets were not affected. Lesions were also rare on the ventral abdomen and inner surfaces of legs, which are the sites preferred by vectors.^11,28^ We supposed that horizontal transmission of viral pathogens between our piglets occurred directly via body contact and via nasal and oral secretions, rather than by indirect transmission via arthropods. Small skin injuries may have served as the main route of entry for SWPV. Stressful regrouping in connection with medical interventions, age-related susceptibility, overcrowding, and other traumatic events is also said to favor the direct animal-to-animal spread of the virus between weaned piglets.^11^

There is no specific therapy for swinepox virus–infected pigs. To control swinepox infections in a farm, minimizing stress factors in combination with the implementation of strict biosecurity measures should be carried out to decrease any risk of transmission. Improvement of hygiene management and insect control measures of the affected farms and, if necessary, antibiotic treatment of pigs with secondary bacterial infections, can be considered as disease control approaches.^15,20,34,47^ Restricted animal movement and hygiene management helped in our case to break the infection chain and to eliminate the disease.

We achieved rapid diagnosis in our case through the use of molecular and TEM methods. Poxvirus detection using the agar gel diffusion precipitation test,^10^ or virus isolation in primary pig kidney cell cultures,^23,34^ is possible but difficult and not suitable for routine diagnosis. Examination of lesion material by negative staining is, despite molecular methods, particularly valuable as a fast frontline technique because TEM allows, with its “open view,” rapid identification of poxviruses and other microorganisms by pattern recognition. From the diagnostic and treatment perspective, the underlying “catch-all principle” of this technique is essential for the fast recognition of an unknown or reemerging agent. In our case, TEM supported the practitioner’s clinical diagnosis of SWPV infection within one hour of receipt of the biopsy in the laboratory.

Routine PCR and sequence analysis on the basis of thymidine kinase analysis confirmed the infection with SWPV in our case. Poxviruses acquire thymidine kinase at or near their time of origin.^16^ As described previously,^14,45^ the conserved swinepox *TK* gene (*SwF8* or *SPV063*) is related to the *TK* genes of other poxviruses (e.g., *CF8* gene of Kenya sheep-1 virus genus *Capripoxvirus*, *SF8* gene of Shope fibroma virus genus *Leporipoxvirus*) and is located within the central core region of the genome in a position similar to that observed for the *TK* gene of VACV (*Orthopoxvirus*, *J2R* gene) and fowlpox virus (*Avipoxvirus*). *TK* genes of most poxviruses, especially those of subclade IIa, are enclosed by homologous *F7* and *F9* genes. Thus, PCR assays, targeting specific *SwF7-* and *SwF8*-*TK* gene sequences, are the tests of choice to routinely identify SWPV at the species and strain level.

Strain differences between wild boar and domestic pig SWPV suggest that the source of infection of healthy domestic piglets is persistently infected subclinical domestic pigs.^22^ In our study, however, the genomic sequence of the whole *TK* sequence of Saxony-Anhalt strains of domestic pigs clustered with the *TK* sequence of the wild boar and not with the sequence of the domestic pigs from Westphalia and Austria. Thus, an epidemiologic origin from wild animals, despite intensive biosecurity measures and complete rearing in isolated pig farms, cannot be ignored. Infections may also be introduced from outside via vectors or a contaminated environment. We had too few data to understand the source of SWPV in our case. Sequence difference has occurred between Asian-lineage SWPV (including Indian and Chinese strains) and European/American-lineage SWPV (including strains from North and South America, Europe, and European Russia), probably caused by the historic domestication and distribution of pigs starting in the 15th century,^22,24,25^ and was confirmed in our study by sequence analysis of the *TK* gene.

The high similarity of the SWPV *TK* gene with *TK* genes from other poxvirus genera was shown in the close relationship of the *TK* genes of poxviruses of clade II. The *TK* gene sequence of *Suipoxvirus* was most closely related to the *TK* sequence of *Capripox*- and *Cervidpoxvirus*, which derived from the genera *Yatapox-* and *Leporipoxvirus*, which was also the ancestor group of *Avipox-* and *Vespertilionpoxvirus*. The genera *Cervidpox*-, *Suipox-*, *Yatapox-*, *Leporipox*-, and *Capripoxvirus* were nominated by ICTV (https://ictv.global/) as CSYLC genera because of the high identity of the central core of their genome. Poxviruses of the genus *Oryzopoxvirus*, which was later also assigned to the CSYLC genera,^2^ and the unclassified porcupinepox virus grouped as an independent branch within the group around *Suipoxvirus*. Regarding this fact, the phylogenetic analysis of poxvirus genera based on the *TK* gene provided a good picture of evolutionary relationships within the family *Poxviridae*, which was also consistent with phylogenetic analysis based on other genes.^1,2,17,24,41^ The study on the *TK* gene of the canarypox virus^4^ confirmed our results. Poxviruses of clade I (*Sciuripox-*, *Orthopox*-, *Centapoxvirus*) were more isolated.

Finally, morphogenesis of SWPV follows the same pattern described in other poxviruses. In light microscopic examination, ballooning degeneration of keratinocytes, intranuclear vacuolation, and formation of eosinophilic intracytoplasmic inclusion bodies, which are sites of virus assembly, give an indication of SWPV infection.^35,46,51^ In SWPV infection, epidermal hyperplasia is less marked compared with other poxviral infections (e.g., avipox infection). Cytopathic effect evidenced by large vacuoles^33,50^ was also not observed in our study, and A and B inclusions were less separated by a well-defined cytoplasmic area.

SWPV assembly, starting with the formation of crescents in virus factories of B inclusions and ending with mature virions in large A inclusions, is essentially the same as that of other poxviruses^8,42^ and of SWPV assembly described in experiments and cell lines.^9,33,50^ Maturation of viruses starts with the enclosure of viroplasm by a crescent membrane and ends with condensation and differentiation of enclosed viroplasm. Although conventional fixation is not optimal, the crescent stages have, as for crescent stages of VACV,^18^ a single bilayered membrane with a smooth concave side and spikes on their convex side. The appearance of a double-layered membrane described from SWPV^9,48^ and from VACV^40^ morphogenesis may result from ultrastructural pictures of transverse incisions through virus stages. Comparable to other poxvirus replications, SWPV crescents in our study never had continuity between cellular intermediate compartments, although proteins from crescents and intermediate compartments are supposed to be of the same origin.^18,54^ Similar to VACV assembly,^8^ looping ends or globular structures at the end of crescents may also play a role in the assembly of immature swinepox virions. These globular structures look like irregular membrane fragments, like those described in fowlpox virus^39^ and in *Orthopoxvirus* assembly.^27,53^ Long cross-striated fibrils associated with immature virus forms have also been noted in the cytoplasm of epidermal cells infected by suipoxvirus,^6^ orthopoxvirus,^12,27,39,40^ or avipoxvirus.^39^ It is questionable whether these cross-striated fibrils seen in the cellular matrix and in close proximity to virus precursors and viroplasm just give rise to the viral envelope^48^ and/or serve as protein deposits associated with viral replication production.^9^ As in VACV assembly,^12,40^ the cytoskeleton may also play an important role during replication and assembly of SWPV, exhibiting considerable crosstalk between virus proteins and both of the cytosolic systems, microtubules, and microfilaments. Interactions between subviral components and cytoskeletal tracks are supposed to help orchestrate virus assembly.^12,38^ Thus, during morphogenesis, viruses may induce rearrangements of cellular cytoskeletal filaments so that they can utilize them as tracks or shove them aside when they are barriers. Perhaps, this may also be a fact in SWPV assembly. Multivesicular bodies, suggested to be involved in protein syntheses and formation of parallel cross-striated fibrils,^6^ were also present in our case with progressive virus production, but were neither associated with these fibrils nor with mature viruses. The fact that crystalloid deposits were also detected in ultrathin sections of our infected piglets, in numerous numbers free in the matrix of B inclusions and less numerous in precursors of A inclusions, supports the idea that these deposits are associated with protein synthesis for virus replication.^53^ The origin of crystalloid deposits from degenerate mitochondria^9,48,50^ because of infection could not be demonstrated in our investigation. The theory that large central laminar aggregates are involved in viral assembly^6^ could also not be supported by our observations.

## Supplemental Material

sj-pdf-1-vdi-10.1177_10406387251366960 – Supplemental material for Diagnostic approach to swinepox virus infection in a German 2-site swine production unit Supplemental material, sj-pdf-1-vdi-10.1177_10406387251366960 for Diagnostic approach to swinepox virus infection in a German 2-site swine production unit by Susanne Richter, Friedrich Schmoll, Daniel Polzer, Christoph Leth, Sandra Revilla-Fernández, Lukas Schwarz, Angelika Auer and Tatjana Sattler in Journal of Veterinary Diagnostic Investigation
