# Supplementary material for: Diagnostic approach to swinepox virus infection in a German 2-site swine production unit
Source: J Vet Diagn Invest. 2025 Aug 30;37(6):882–90. doi: 10.1177/10406387251366960 (PMC12398465; doi:10.1177/10406387251366960)
Supplement: Supplementary file 1 [file sj-pdf-1-vdi-10.1177_10406387251366960.pdf]

JVDI: Supplemental material

Richter S, et al. Diagnostic approach to swinepox virus infection in a German 2-site swine production unit

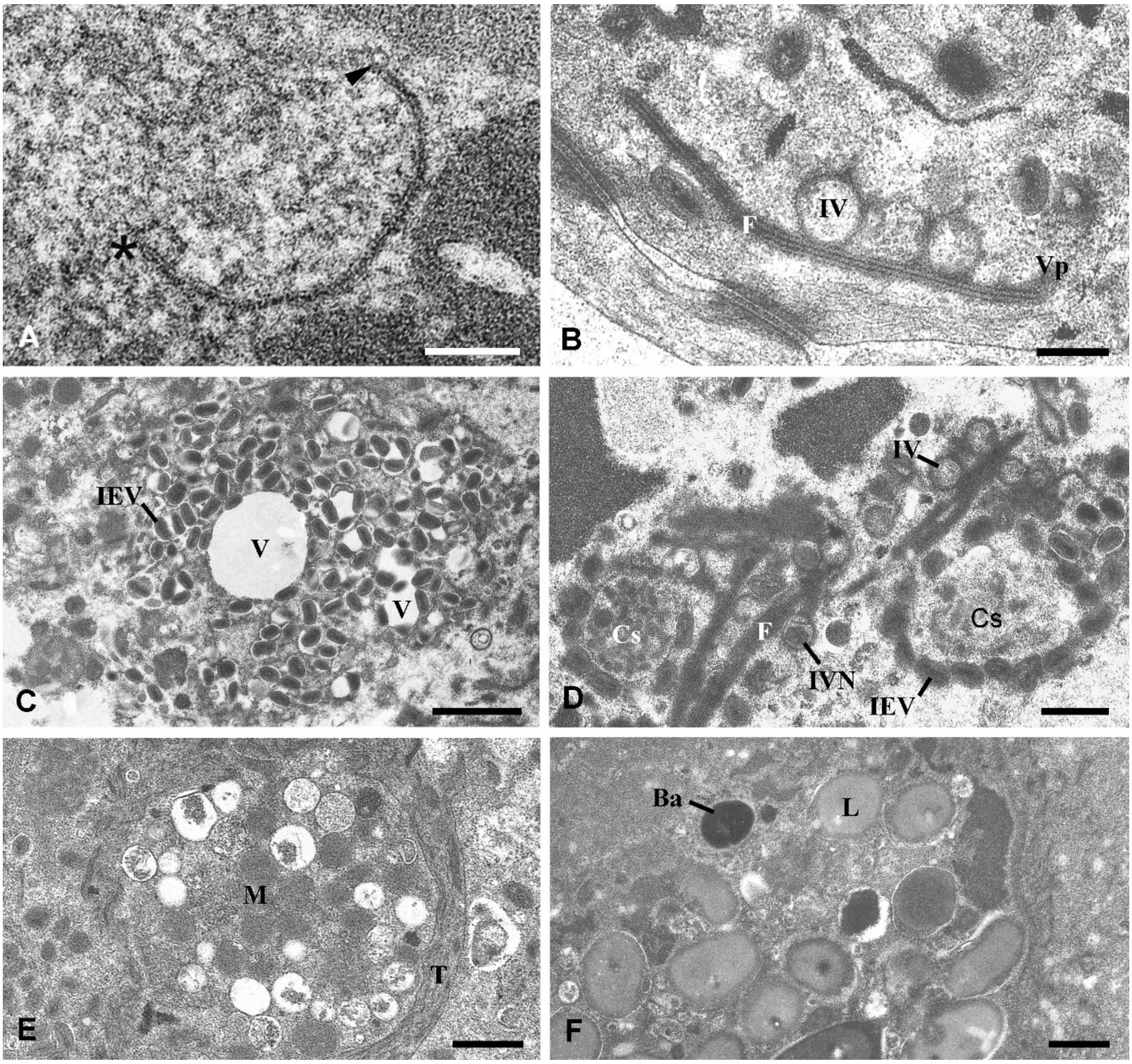

Supplemental Figure 1. Further aspects of swinepox virus assembly. Transmission electron micrographs. **A.** The virus crescent, a single membrane bilayer, with a "spicule" layer at the convex surface and a globular vesicle (arrowhead) and loop (asterisk) at the end, revealed no continuity between cellular intermediate compartments. Bar = 100 nm. **B.** Spherical immature virion (IV) and viroplasm (Vp) in close contact with parallel, cross-

striated fibrils (F). Bar = 250 nm. **C.** Young A-type inclusion body with one large and few smaller lipid vacuoles (V) surrounded by numerous intracellular enveloped virion (IEV) stages. Bar = 1 μm. **D.** B-type inclusion with groups of parallel, cross-striated fibrils (F) and immature viral particles with uniform viroplasm (IV) and with condensed viroplasm (IVN). Formation of early A-type inclusions with IEVs enclosing a cytoplasmic area with crystalloid deposits (Cs), arranged in a honeycomb pattern. Bar = 500 nm. **E.** Multivesicular bodies (M), sometimes membrane-bound, are frequent in cells with virus development. Tonofibrils (T) at the cell plasma periphery are characteristic of stratified squamous epithelium. Bar = 500 nm. **F.** Lipid globules (L) and bacteria (Ba) from secondary infections within the cytoplasm of epidermal cells. Bar = 500 nm.
